# The relationship between meat consumption and frailty among community-residing older adults in Nanjing municipality, China

**DOI:** 10.3389/fnut.2026.1955451

**Published:** 2026-10-06

**Authors:** Zhao Dong, Qing Ye, Caihong Hu, Yunting Xu, Guofeng Ao, Luyan Yang, Fengli Li, Shifen Jia, Yaqing Xiong, Yu Dou

**Affiliations:** 1 Nanjing Vocational Health College, Nanjing, China; 2 Nanjing Medical University Affiliated Nanjing Municipal Center for Disease Control and Prevention, Nanjing, China; 3 Jiangsu Province Official Hospital, Nanjing, China; 4 Geriatric Hospital of Nanjing Medical University, Nanjing, China

**Keywords:** aging, Chinese, frailty, meat, older adults

## Abstract

**Background:**

Frailty is a common complaint in older adults. Although the world is experiencing a rapid process of aging, the relationship between meat consumption and frailty was not well-documented. This study aimed to examine the association of meat consumption with frailty among older adults in China.

**Methods:**

In this cross-sectional study, participants were randomly chosen from community-dwelling residents aged 60 years or above in Nanjing municipality, China, in 2023. Frailty, assessed using Groningen Frailty Indicator, was the outcome variable. Meat consumption frequency, measured using a validated food frequency questionnaire, was dichotomized as independent variable based on official recommendations by China Nutrition Society. Mixed-effects logistic regression models were introduced to compute odds ratios (ORs) and 95% confidence intervals (CIs) for examining associations between meat consumption and frailty.

**Results:**

Among 5,484 participants, frailty prevalence was 16.9% (95% CI = 15.9, 17.9). Significant difference in frailty prevalence was observed among participants by age (the younger-old *vs*. the middle-old *vs*. the oldest-old: 13.2% vs.17.9% vs. 32.8%), sex (men vs. women: 15.8% vs. 17.9%) or area (urban vs. sub-urban: 15.1% vs. 18.1%). After adjustment for potential confounders, participants who reached recommendation of meat consumption were less likely to have frailty (OR = 0.70, 95% CI = 0.60, 0.81) relative to those who consumed meat below the recommended level. Moreover, when data were analyzed by age, sex or residential area, the relationship between meat intake and frailty remained significant among all the subgroups with an exception of that it became non-significant for the oldest-old adults.

**Conclusions:**

A inverse relationship may exist between meat intake and frailty among community-dwelling older individuals in regional China. This study has particular significance of public health for building the healthy-aging society, suggesting that inadequate intake of meat may be a risk factor of frailty for community-dwelling older adults.

## Introduction

Frailty, a common clinical syndrome, is highly prevalent among older adults and markedly affects their life quality (1–3). It has been documented that the global prevalence rate of frailty was approximately 12.0% among community-dwelling adults aged 50 years or above (4). Meanwhile, its incidence rate was about 43.4/1,000 person-years among people aged 60 years and older worldwide (5). For China, the country with the most populous older residents in the world, it witnessed a prevalence of 10.1% and an incidence rate of 60.6/1,000 person-years for frailty among community-dwelling individuals aged 60 years or above (6, 7). Clearly, frailty is an age-dependent health-related condition, implying that its prevalence rate will continue to rise in the context of rapid population aging worldwide (8, 9). Therefore, frailty imposes a substantial disease burden on older adults and the entire global community.

Usually, frailty is a fluctuating state that involves improvement and progression dynamically for older adults (9–11). This means that frailty is potentially controllable and even preventable (9–11). Logically, for effective control or prevention of frailty, one of the first important things is to identify those frailty-associated factors that can be modified. Fortunately, several modifiable factors of frailty have been identified among older adults, including lifestyle and behaviors (12–14). Among those frailty-associated lifestyle and behaviors, meat consumption was least investigated so far (13, 14). Meat can provide necessary energy, high quality protein and essential amino acids, and thus is vital for maintaining human health, particularly for older people (15–17). To better understand the modifiable factors of frailty in daily life, it is in need to further explore the association of meat consumption, a typical eating behavior, with frailty among older adults.

Undoubtedly, the world has been experiencing population aging rapidly, particularly in developing countries like China (8). Today, China has the largest number of people aged 60 years and older in the world (18). Moreover, the prevalence of frailty is increasing among older adults in China, implying that China will witness a much greater burden of frailty in future (19, 20). Hence, there is a unique opportunity for investigating frailty and its associated factors such as meat consumption in China. Considering that: (1) frailty is an age-dependent but controllable health-related state, (2) population aging has been occurring globally and rapidly, and (3) evidence remains scarce on the association between meat consumption and frailty (1–3), a community-based study was developed to specifically examine the relationship between meat consumption and frailty among community-dwelling older adults in Nanjing municipality, China.

## Methods

### Study design and participants

Data analyzed in this study were derived from a cross-sectional population study, which was conducted among community-residing older adults in Nanjing municipality of China in 2023 (21–23). The study city had approximately 9.5 million regularly registered residents (22.0% of them aged 60 years or above) and 12 administrative districts in the survey year (24). The participants, study design, and data collection have been described in detail elsewhere (21–23). Briefly, only those individuals were eligible to take part in this investigation, if they were: (1) the regularly registered community-dwelling residents in Nanjing, (2) 60 years older or above, (3) not having literacy or/and cognitive problems, (4) able to do routine activities in daily living, and (5) not experiencing acute infectious diseases (21–23). The estimated sample size was approximately 6,000 for the broad cross-sectional survey (21–23). Considering the available prevalence of frailty (10.1%) among community-dwelling older residents aged 60 years or above in China (6), such a large sample size was sufficient for investigating the association of meat consumption with frailty in the present study.

A multistage sampling method was employed to randomly select participants from all the 12 administrative districts in Nanjing (21–23). Firstly, two streets/townships were chosen from each district. Secondly, one administrative residence community was selected from each determined street/township, resulting in a total of 24 communities involved. Thirdly, the number of participants (*N* = 250) that would be recruited from each chosen community was computed by 6,000 [overall sample size] dividing by 24 [the number of communities selected]. Finally, using the household list, eligible older adults were randomly determined from each involved community based on the age- and sex-proportion of older residents in Nanjing (24). Actually, 6,002 eligible participants were selected in this study. Figure 1 displays the flow chart of participant's determination.

**Figure 1:**
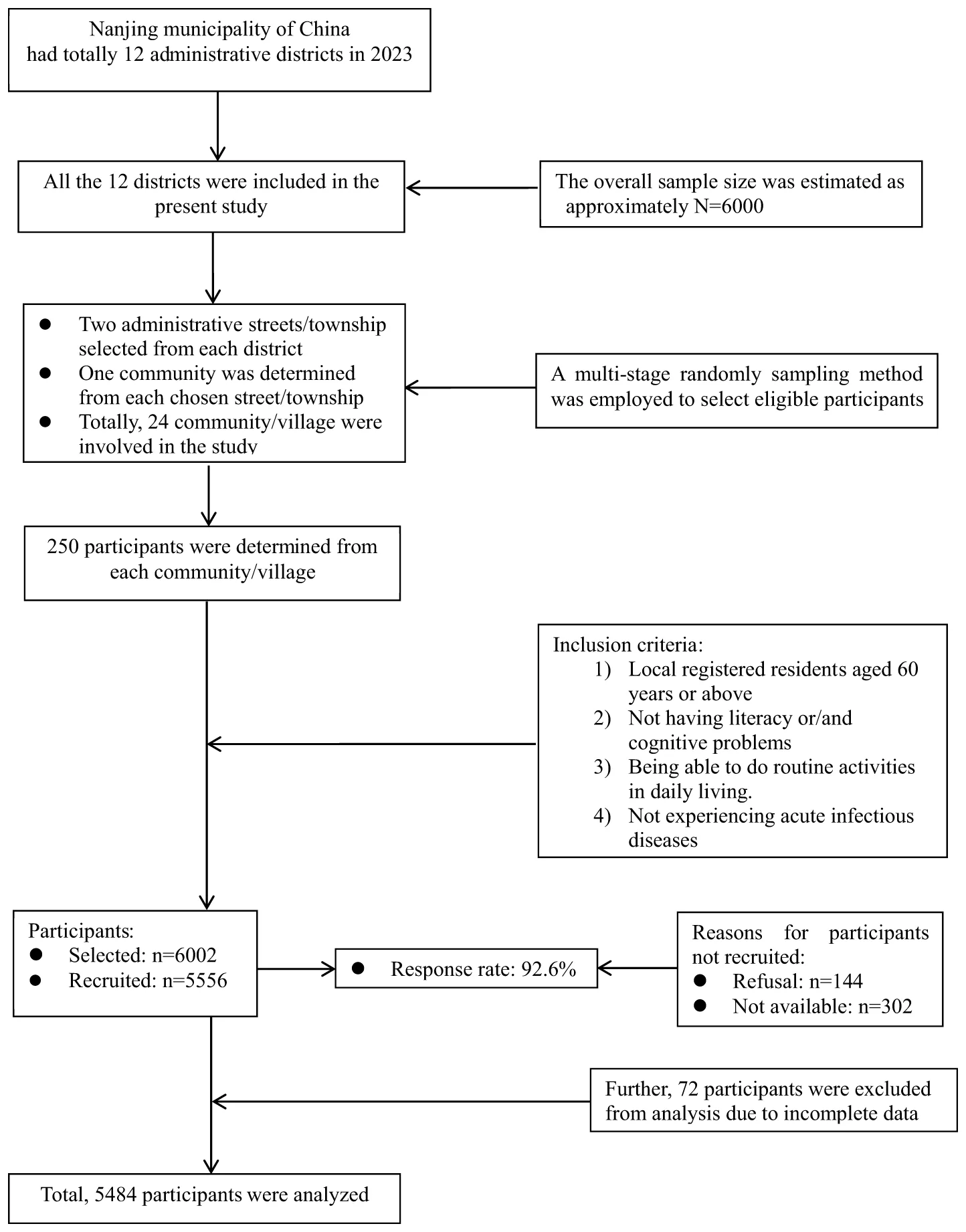
Flow chart of participant's determination in this study.

Each participant provided written informed consent before the survey. The present study was reviewed and approved by the Ethics Committee of Nanjing Medical University Affiliated Nanjing Municipal Center for Disease Control and Prevention. The study methods fully comply with relevant recommendations in the Helsinki Declaration.

### Data collection

In 2018, China National Center for Non-communicable Disease Control and Prevention (CCNDC) recommended a standardized procedure of data collection, measurement instruments, and variable definitions in the *Scheme of the Chinese Chronic Non-Communicable Disease (NCD) and Risk Factor Surveillance* (25). The CCNDC released this *scheme* with the main purpose to warrant a good comparability across studies on NCDs and associated factors in China (25). Definitely, the field survey procedure, definitions and classifications of measures employed in the present study strictly aligned with those recommended in the *scheme* by CCNDC (25). Each selected participant was invited to take part in a face-to-face interview for data collection at the local community health service center (26). Participants self-reported the information on their socio-demographic characteristics, behaviors and lifestyle, common NCDs, sleep quality, frailty, and depressive symptoms (21–23). Additionally, their anthropometric metrics and lipid profiles were objectively assessed (25). Body weight was measured to the nearest 0.1 kilogram, while body height was recorded to the nearest 1.0 centimeter (25). Each of them was assessed twice, and the mean values were used to calculate body mass index (BMI) (25). Meanwhile, lipid profiles (triglyceride, cholesterol, high-density and low-density lipoproteins) were tested with fasting venous blood sample at qualified laboratories (25).

### Study variables

#### Outcome variable

Frailty was the outcome variable. It was assessed using the validated Chinese version of Groningen Frailty Indicator (GFI-CHN) (26, 27). Steverink and colleagues developed the GFI, with an optimal cutoff of 4 points, for identifying frailty in older adults (28, 29). The GFI-CHN performed well for screening frailty among older adults in China, showing a good internal consistency, test-retest reliability, criterion validity against a 37-health-deficit frailty index (FI), and concurrent validity against Fried's frailty phenotype (26, 27). In this study, participants were classified as “without frailty (GFI < 4)” or “with frailty (GFI≥4)” for analysis (27–29).

#### Independent variable

Meat consumption was the independent variable. It referred to intake of either red or white meat, and was measured using a validated Chinese version of Food Frequency Questionnaire (FFQ) (30). In 2016, China Nutrition Society (CNS) released an update of dietary guidelines for older Chinese people (31). In this food consumption guidelines, CNS did not recommend intake level for red and white meat separately, but just combined them together as meat consumption (31). Moreover, only adequate frequency of meat consumption (times per week) was recommended for older Chinese residents by CNS (31).

Using the FFQ, participants were asked to self-report how often to consume red and white meat, separately, in last 12 months under a typical circumstance (30). Next, the frequency of red and white meat consumption was, respectively, converted as times per week. Then, the combined weekly frequency of both red and white meat intake was calculated for each participant. Subsequently, based on this CNS recommended consumption frequency of meat for older adults, participants were dichotomized as “recommendation not reached (< 7 times/week)” or “recommendation reached (≥7 times/week)” (31).

#### Covariates

Classical covariates of frailty were controlled for in multivariate analyses. They were socio-demographic characteristics, behaviors and lifestyle, common physical NCDs, depressive symptoms, body weight status, and lipid profiles. Socio-demographic attributes included: age [60–69 years (the younger-old), 70–79 years (the middle-old), or 80+ years (the oldest-old)], sex (men or women), residence area (urban or sub-urban), educational attainment [ ≤ 6 (primary school or less), 7–12 (middle school), or 13+ years of schooling (college or above)], marital status (single or having a spouse/partner), occupation before retirement (domestic worker, service/sales persons, industrial worker, office worker, academic/research staff, or self-employed people) (18, 32), and healthcare insurance coverage (employee medical insurance scheme, or resident medical insurance scheme (33, 34).

Behaviors and lifestyle referred to physical activity (PA), sedentary behavior (SB), vegetable intake, drinking, smoking, and sleep. PA and SB were assessed with the Chinese version of International Physical Activity Questionnaire (35). Participants were classified as engaging in “insufficient (< 150 min/7 days)” or “sufficient (≥150 min/7 days)” PA based on the sum of moderate and doubled vigorous PA time in last seven days, and “prolonged SB (≥2 h/day)” or “shortened SB (< 2 h/day)” according to average daily screen-viewing time, respectively (36). Moreover, participants were categorized as “drinkers” or “non-drinkers,” and “smokers” or “non-smokers,” separately, using definitions adopted from those recommended in the *scheme* for NCDs survey by CCNDC (25). Vegetable intake was measured with the same FFQ used to assess meat consumption (30). Participants were then divided into “recommendation not reached (< 14 times/week)” or “recommendation reached (≥14 times/week)” according to the vegetable intake frequency recommended for older Chinese adults by CNS in 2016 (31). Additionally, sleep quality was assessed with the validated Chinese version of Pittsburgh Sleep Quality Index (PSQI-CHN) (37, 38). The 7-point cutoff score was used to categorize participants as having “poor (PSQI>7)” or “good (PSQI ≤ 7)” sleep quality (37, 38).

Associated chronic physical diseases included hypertension, diabetes, coronary heart disease (CHD), stroke, chronic obstructive pulmonary disease (COPD), chronic kidney disease (CKD), and cancer. The history of each selected chronic disease was self-reported by participants and classified as “Yes” or “No” (25). Depressive symptoms were determined with the validated Chinese version of Patient Health Questionnaire-9 (PHQ-9), and categorized as “positive (PHQ score ≥5)” or “negative (PHQ score < 5)” for each participant (39, 40).

As for the status of participant's body weight, based on the Chinese-adult-sensitive cutoffs, it was classified into: “underweight (BMI < 18.5),” “normal weight (BMI = 18.5, 23.9),” “overweight (BMI = 24.0, 27.9),” or “obesity (BMI ≥ 28 kg/m^2^)” (41). Additionally, participants were recorded as having “normal lipid profile” only when all of triglyceride, cholesterol, high-density and low-density lipoproteins were simultaneously at normal level, otherwise recorded as having “abnormal lipid profile” (25).

### Statistical analysis

Chi-square tests were conducted to compare differences in the distribution (%) of meat consumption and frailty among participants by socio-demographic characteristics, separately. Then, two mixed-effects logistic regression models were employed to calculate odds ratios (ORs) and 95% confidence intervals (CIs) for examining associations between meat consumption and frailty. Model 1 was a univariate analysis with meat consumption as the single explanatory variable and survey community as the random effect. Model 2 was a multivariate regression analysis with adjustment for age (where applicable), sex (where applicable), residence area (where applicable), educational attainment, marital status, occupation, healthcare insurance, body weight status, PA, SB, drinking, smoking, vegetable consumption, sleep quality, depressive symptoms, hypertension, diabetes, CHD, stroke, CKD, COPD, cancer, and fasting blood lipid status in addition to those included in Model 1. The significance level was set as *p* < 0.05 (two-sided). Data analysis was conducted with R (version 4.4.3, R Foundation, Vienna, Austria).

## Results

There were 6,002 participants initially selected. Then, 5,556 of them were recruited successfully. Among those 446 participants not recruited, 144 refused to take part in the study and 302 were unavailable. Finally, 5,484 were analyzed, as 72 were excluded due to incomplete data. The differences in age, sex, or residential area were not statistically significant between participants not included and included in the present study.

Table 1 shows the characteristics of participants analyzed in this study by sex. For overall participants, the mean age (standard deviation) was 69.7 (6.8), and the proportions of men and urban residents were 49.0% and 39.1%, separately. Moreover, 57.6%, 31.2%, and 11.2% of participants were the younger-old, the middle-old, and the oldest-old adults, respectively. Additionally, 5.5% of participants obtained an education of college level or above, while only 1.6% were underweight with BMI < 18.5 kg/m^2^.

**Table 1:** Selected characteristics of participants among community-residing older adults aged 60 years or above by sex in this study (*N* = 5,484).

Characteristics			% (***n***) of participants	
		Total	Men	Women
Overall		5,484	49.0 (2,687)	51.0 (2,797)
Age (years)				
	60–69	57.6 (3,157)	57.3 (1,540)	57.8 (1,617)
	70–79	31.2 (1,711)	31.5 (846)	30.9 (865)
	80+	11.2 (616)	11.2 (301)	11.3 (315)
Residing area^a^				
	Urban	39.1 (2,144)	39.4 (1,060)	38.8 (1,084)
	Sub-urban	60.9 (3,340)	60.6 (1,627)	61.2 (1,713)
Educational attainment (years of schooling)				
	0–6	49.6 (2,721)	38.9 (1, 044)	60.0 (1,677)
	7–12	44.9 (2,465)	53.8 (1,447)	36.4 (1, 018)
	13+	5.5 (298)	7.3 (196)	3.6 (102)
Occupation^b^				
	Domestic worker	19.6 (1,075)	15.7 (422)	23.3 (653)
	Industrial worker	42.4 (2,327)	42.1 (1,131)	42.8 (1,196)
	Service/sales worker	6.2 (338)	6.1 (165)	6.2 (173)
	Office worker	8.1 (443)	9.9 (264)	6.4 (179)
	Academic/research staff	7.0 (384)	8.6 (232)	5.4 (152)
	Self-employed	16.7 (917)	17.6 (473)	15.9 (444)
Marital status				
	Single	13.8 (758)	10.7 (288)	16.8 (470)
	Having a spouse/partner	86.2 (4,726)	89.3 (2,399)	83.2 (2,327)
Healthcare insurance^c^				
	EMI	56.6 (3,104)	51.6 (1,387)	61.4 (1,717)
	RMI	43.4 (2,380)	48.4 (1,300)	38.6 (1,080)
Body weight status^d^				
	BMI < 18.5 kg/m^2^	1.6 (87)	1.7 (45)	1.5 (42)
	18.5 ≤ BMI < 24.0 kg/m^2^	41.2 (2,259)	41.8 (1,124)	40.6 (1,135)
	24.0 ≤ BMI < 28.0 kg/m^2^	41.8 (2,293)	42.1 (1,130)	41.6 (1,163)
	BMI ≥ 28.0 kg/m^2^	15.4 (845)	14.4 (388)	16.3 (457)

a, Urban and sub-urban areas were defined based on the official classifications released by China national bureau of statistics. b, Occupation referred to job before retirement, and was classified for each participant based on recommendations by China national center for chronic non-communicable disease prevention and control. c, Health insurance: EMI, employee medical insurance scheme, RMI, resident medical insurance scheme. d, Body weight status was categorized based on cutoffs of body mass index (BMI) recommendations for Chinese adults.

Table 2 illustrates the distribution of meat consumption among participants. Overall, 62.6% of participants reached the CNS-recommended level of meat consumption. The proportion of participants who reached meat intake recommendation was significantly different by each of age, sex, residential area, educational attainment, occupation, marital status, and health insurance, but not by body weight status. Specifically, the oldest-old adults, women, and sub-urban residents consumed meat less frequently compared to their corresponding counterparts, separately.

**Table 2:** The distribution of meat consumption among community-residing older adults aged 60 years or above by selected characteristics in this study (*N* = 5,484).

Characteristics			% (*n*) of participants meeting the recommendation of meat consumption^a^		*χ^2^*	*p* value
		Total	No	Yes		
Overall		5,484	37.4 (2, 049)	62.6 (3,435)		
Age (years)						
	60–69	57.6 (3,157)	36.7 (1,158)	63.3 (1,999)		
	70–79	31.2 (1,711)	36.8 (629)	63.2 (1,082)	7.93	0.019
	80+	11.2 (616)	42.5 (262)	57.5 (354)		
Sex						
	Men	49.0 (2,687)	35.5 (953)	64.5 (1,734)	8.09	0.004
	Women	51.0 (2,797)	39.2 (1,096)	60.8 (1,701)		
Residing area^b^						
	Urban	39.1 (2,144)	23.9 (513)	76.1 (1,631)	271.55	< 0.001
	Sub-urban	60.9 (3,340)	46.0 (1,536)	54.0 (1,804)		
Educational attainment (years of schooling)						
	0–6	49.6 (2,721)	44.3 (1,205)	55.7 (1,516)		
	7–12	44.9 (2,465)	31.2 (770)	68.8 (1,695)	115.23	< 0.001
	13+	5.5 (298)	24.8 (74)	75.2 (224)		
Occupation^c^						
	Domestic worker	19.6 (1,075)	34.6 (372)	65.4 (703)		
	Industrial worker	42.4 (2,327)	46.2 (1,074)	53.8 (1,253)		
	Service/sales worker	6.2 (338)	23.4 (79)	76.6 (259)		
	Office worker	8.1 (443)	24.6 (109)	75.4 (334)	157.75	< 0.001
	Academic/research staff	7.0 (384)	29.2 (112)	70.8 (272)		
	Self-employed	16.7 (917)	33.0 (303)	67.0 (614)		
Marital status						
	Single	13.8 (758)	48.5 (368)	51.5 (390)	47.02	< 0.001
	Having a spouse/partner	86.2 (4,726)	35.6 (1,681)	64.4 (3, 045)		
Healthcare insurance^d^						
	EMI	56.6 (3,104)	45.9 (1,424)	54.1 (1,680)	221.48	< 0.001
	RMI	43.4 (2,380)	26.3 (625)	73.7 (1,755)		
Body weight status^e^						
	BMI < 18.5 kg/m^2^	1.6 (87)	42.5 (37)	57.5 (50)		
	18.5 ≤ BMI < 24.0 kg/m^2^	41.2 (2,259)	37.7 (851)	62.3 (1,408)	1.41	0.703
	24.0 ≤ BMI < 28.0 kg/m^2^	41.8 (2,293)	37.2 (853)	62.8 (1,440)		
	BMI ≥ 28.0 kg/m^2^	15.4 (845)	36.4 (308)	63.6 (537)		

a, Participants who consumed ≥7 times/week were defined as meeting the consumption recommendation for Chinese older adults released by Chinese Nutrition Society in 2016. Otherwise, they were defined as not meeting the consumption recommendation. b, Urban and sub-urban areas were defined based on the official classifications released by China National Bureau of Statistics. c, Occupation referred to job before retirement, and was classified for each participant based on recommendations by China National Center for Chronic Non-communicable Disease Prevention and Control. d, Healthcare insurance: EMI, employee medical insurance scheme, RMI, resident medical insurance scheme. e, Body weight status was categorized based on cutoffs of body mass index (BMI) recommendations for Chinese adults.

Table 3 presents frailty prevalence among participants by characteristics. Among overall participants, GFI-assessed frailty prevalence was 16.9% (95% CI = 15.9, 17.9). As expected, the prevalence of frailty was age-dependent. Among the younger-old, the middle-old and the oldest-old adults, the prevalence was 13.2%, 17.9% and 32.8%, respectively (*p* < 0.001). Moreover, frailty prevalence was higher in women (17.9%) relative to men (15.8%), and higher among sub-urban individuals (18.1%) compared to the urban residents (15.1%) (*p* < 0.05 for each). However, notably, the prevalence of frailty did not differ among participants stratified by body weight status.

**Table 3:** The prevalence of frailty among community-dwelling adults aged 60 years or above by selected characteristics in this study (*N* = 5,484).

Characteristics			% (*n*) of participants experiencing frailty^a^		*χ^2^*	*p* value
		Total	Non-frail	Frail		
Overall		5,484	83.1 (4,558)	16.9 (926)		
Age (years)						
	60–69	57.6 (3,157)	86.8 (2,739)	13.2 (418)		
	70–79	31.2 (1,711)	82.1 (1,405)	17.9 (306)	142.16	< 0.001
	80+	11.2 (616)	67.2 (414)	32.8 (202)		
Sex						
	Men	49.0 (2,687)	84.2 (2,262)	15.8 (425)	4.29	0.038
	Women	51.0 (2,797)	82.1 (2,296)	17.9 (501)		
Residing area^b^						
	Urban	39.1 (2,144)	84.9 (1,821)	15.1 (323)	8.31	0.004
	Sub-urban	60.9 (3,340)	81.9 (2,737)	18.1 (603)		
Educational attainment (years of schooling)						
	0–6	49.6 (2,721)	80.4 (2,187)	19.6 (534)		
	7–12	44.9 (2,465)	85.9 (2,117)	14.1 (348)	28.96	< 0.001
	13+	5.5 (298)	85.2 (254)	14.8 (44)		
Occupation^c^						
	Domestic worker	19.6 (1,075)	85.2 (887)	17.5 (188)		
	Industrial worker	42.4 (2,327)	80.8 (1,880)	19.2 (447)		
	Service/sales worker	6.2 (338)	86.4 (292)	13.6 (46)	22.08	0.001
	Office worker	8.1 (443)	85.3 (378)	14.7 (65)		
	Academic/research staff	7.0 (384)	85.7 (329)	14.3 (55)		
	Self-employed	16.7 (917)	86.4 (792)	13.6 (125)		
Marital status						
	Single	13.8 (758)	73.0 (553)	27.0 (205)	64.369	< 0.001
	Having a spouse/partner	86.2 (4,726)	84.7 (4, 005)	15.3 (721)		
Healthcare insurance^d^						
	EMI	56.6 (3,104)	80.9 (2,511)	19.1 (593)	25.09	< 0.001
	RMI	43.4 (2,380)	86.0 (2, 047)	14.0 (333)		
Body weight status^e^						
	BMI < 18.5 kg/m^2^	1.6 (87)	78.2 (68)	21.8 (19)		
	18.5 ≤ BMI < 24.0 kg/m^2^	41.2 (2,259)	82.9 (1,873)	17.1 (386)	2.09	0.554
	24.0 ≤ BMI < 28.0 kg/m^2^	41.8 (2,293)	83.2 (1,907)	16.8 (386)		
	BMI ≥ 28.0 kg/m^2^	15.4 (845)	84.0 (710)	16.0 (135)		

a, Non-frail was defined as the score of Groningen Frailty Indicator < 4, while frail was determined as the score ≥ 4. b, Urban and sub-urban areas were defined based on the official classifications released by China National Bureau of Statistics. c, Occupation referred to job before retirement, and was classified for each participant based on recommendations by China National Center for Chronic Non-communicable Disease Prevention and Control. d, Healthcare insurance: EMI, employee medical insurance scheme, RMI, resident medical insurance scheme. e, Body weight status was categorized based on cutoffs of body mass index (BMI) recommendations for Chinese adults.

Table 4 elucidates the association between recommended level of meat consumption and frailty among older adults in the study. After control for confounders, participants who reached recommendation of meat consumption were less likely to have frailty (OR = 0.70, 95% CI = 0.60, 0.81) than those who consumed meat below the recommended level. When data were analyzed by stratification of age, sex, or residential area, the relationship between meat intake and frailty remained significant among all the subgroups of participant with an exception of that it became non-significant for the oldest-old adults.

**Table 4:** The association between meeting recommendation of meat consumption and frailty among community-residing older adults aged 60 years or above in this study (*N* = 5,484).

Characteristics		Recommendation of meat consumption ^b^	OR (95% CI) for experiencing frailty ^a^		
			% (n/N) of participants with frailty ^c^	Model 1^c^	Model 2^d^
Overall					
		Not meet	21.8 (446/2,049)	1	1
		Meeting recommendation	14.0 (480/3,435)	0.58 (0.51, 0.67)	0.70 (0.60, 0.81)
Age (years)								
	60–69
		Not meet	18.0 (209/1,158)	1	1
		Meeting recommendation	10.5 (209/1,999)	0.53 (0.43, 0.65)	0.65 (0.52, 0.81)
	70–79
		Not meet	24.5 (154/629)	1	1
		Meeting recommendation	14.0 (152/1,082)	0.50 (0.39, 0.65)	0.56 (0.43, 0.74)
	80+
		Not meet	31.7 (83/262)	1	1
		Meeting recommendation	33.6 (119/354)	1.09 (0.78, 1.54) ^e^	1.45 (0.97, 2.18) ^e^
Sex					
	Men
		Not meet	19.9 (190/953)	1	1
		Meeting recommendation	13.6 (235/1,734)	0.63 (0.51, 0.78)	0.71 (0.56, 0.90)
	Women
		Not meet	23.4 (256/1,096)	1	1
		Meeting recommendation	14.4 (245/1,701)	0.58 (0.45, 0.67)	0.69 (0.55, 0.85)
Residing area					
	Urban
		Not meet	23.4 (120/513)	1	1
		Meeting recommendation	12.4 (203/1,631)	0.47 (0.36, 0.60)	0.52 (0.39, 0.69)
	Sub-urban
		Not meet	21.28 (326/1,536)	1	1
		Meeting recommendation	15.4 (277/1,804)	0.67 (0.56, 0.80)	0.79 (0.65, 0.95)

a, Frailty was defined as a score ≥ 4 assessed with the Groningen Frailty Indicator. b, Participants who consumed ≥ 7 times/week were defined as meeting the consumption recommendation for Chinese older adults released by Chinese Nutrition Society in 2016. Otherwise, they were defined as not meeting the consumption recommendation. c, Model 1 was a univariate logistic regression analysis with meat consumption as the single independent variable and consideration of community as the potential random-effects. d, Model 2 was a multivariate logistic regression analysis with control for age (where applicable), sex (where applicable), residence area (where applicable), educational attainment, marital status, occupation, healthcare insurance, body weight status, physical activity, sedentary behavior, drinking, smoking, vegetable consumption, sleep quality, depressive symptoms, hypertension, diabetes, coronary heart disease, stroke, chronic kidney disease, COPD, cancer, and fasting blood lipid status, in addition to those included in Model 1. e, non-significant.

A multistage sampling approach was employed to randomly determine participants with community as the smallest sampling unit in this study. This implies potential community-level clustering effect may exist among participants. Therefore, the potential community-level clustering effect was examined, and mixed-effects models were introduced to analyze the data. It was found that the community-level variance and intraclass correlation coefficient (ICC) among participants were 1.3 and 0.06, respectively. This reflects that the community-level random effect size was pretty small, suggesting that there was no significant difference in participants selected from the 24 communities in the entire municipality.

## Discussion

This population-based study aimed primarily to examine the relationship between meat consumption and frailty among community-dwelling older adults aged 60 years and older in regional China. It was observed that older people who consumed meat at the CNS-recommended level were less likely to experience frailty compared to those consumed meat below the recommended level in Nanjing municipality of China. Moreover, such a inverse relationship was also observed among participants stratified by age, sex, or area with an exception of the oldest-old individuals.

According to a recent systematic review, very few investigations were available regarding the relationship between diet-related behavioral factors and frailty in older adults worldwide, particularly in China (14). Among the existing studies using meat consumption as the single independent variable, one was from Japan, the remaining two were conducted in China (42–44). All of these three studies did not differentiate red and white meat from each other, and consistently documented that meat intake was inversely associated with frailty among older adults (42–44). In the Japanese study, meat intake was treated as a quantitative variable (g/day) with one standard deviation as a measure of amount in analysis (42). In the two Chinese studies, an integrated index, the dietary diversity score (DDS), was treated as the primary independent variable, and meat consumption frequency as a secondary explanatory measure (43, 44). Moreover, meat intake frequency was used as ordinal classifications (“Almost every day,” “At least once a week,” “At least once a month,” “Occasionally,” and “Rarely or never”), or dichotomized with an unjustified cutoff point of 14 times/week in the Chinese studies (43, 44). For our study, meat consumption was recorded as weekly frequency and dichotomized using the official cutoff value (7 times per week) recommended by CNS (31). Although differences existed in the measurement and classification of meat intake among these aforementioned studies and ours, the consistent findings were observed (42–44). This implies that our study provides further population-level evidence on the inverse relationship between meat intake and frailty based on data from locally representative community-dwelling older adults.

For older adults, loss of muscle mass and/or strength is one of the key contributors to frailty (45), which may suggest the potential explanations for the inverse association of meat intake with frailty. First, it has been reported that adequate intake of protein was inversely associated with frailty among older adults (46). Clearly, meat contains proteins with high biological values, and thereby can increase muscle synthesis and consequently maintain muscle strength and function (47, 48). Second, meat is also rich in several micronutrients, including iron, selenium, zinc, and vitamin B12 (49). Importantly, these micronutrients contained in meat are with highly bioavailable forms, and are easy to be absorbed and utilized (49). Meaningfully, these nutrients are of help for maintaining strong vitality and muscle strength, and thus can also, at least partly, explain why intake of meat is inversely associated with frailty.

As for the non-significant relationship between meat intake and frailty among the oldest-old adults in this study, it may be due to the following plausible reasons. Typically, with age advancing, the older adults need energy to a reduced level, and may experience loss of appetite, gastrointestinal problems, and commorbid chronic diseases, which can result in a reduction in protein utilization (47, 50–52). This implies that, even if the same level of meat consumed, the metabolic efficacy may be lower for the oldest-older adults than that for younger-old individuals. Moreover, all these age-dependent unfavorable conditions may also contribute to a different consumption pattern of protein-rich meat among the oldest-old adults (53). In the present study, the average frequency of meat intake was 4.22, 4.12, and 3.80 times/week among the younger-, the middle-, and the oldest-older residents (*P* < 0.01), respectively, demonstrating that the oldest-older adults consumed meat least frequently. Importantly, compared to the younger-older people, the oldest-older adults may also intake lower amount of meat each time due to loss of appetite, gastrointestinal problems, change in food preference (47, 50–52). However, no data were available on the intake amount of meat, which did not allow us to make a further analysis. Notably, the specific association between meat intake and frailty among the oldest-older adults is worthy of deep investigation in future.

The world has been witnessing a rapid process of population aging with an unprecedented pace (8). This means that frailty, an age-dependent chronic condition, will impose increasingly considerable disease burden upon the societies and families worldwide in future (19, 20). Therefore, to identify the modifiable factors is important for frailty management at both population and individual level. Those modifiable factors can be used as markers to screen older adults at high risk of frailty, and even used as intervention approaches against frailty if the potential causality confirmed. The present study investigated the association of meat consumption, a typically common dietary behavior, with frailty among older residents aged 60 years or above in regional China. Interesting and meaningful findings were examined that reaching the recommended level of meat intake was inversely associated with the risk of frailty not only among overall older adults, but also within participants stratified by age, sex, or residential area with just one exception of non-significance observed in the oldest-older people. This probably implies that the inverse association between meat consumption and frailty may broadly hold for the general local older residents in Nanjing, China.

This study has several strengths. First, community-dwelling individuals were randomly determined from general older residents, who were of representativeness of the overall 1.95 million registered older inhabitants in Nanjing (24). Second, a specific instrument, the validated Chinese version of GFI, was employed to measure frailty. Third, meat consumption was also assessed using a validated population-sensitive FFQ. Fourth, the latest official recommendation of meat consumption by China Nutrition Society was used to evaluate meat intake level. Fifth, classical confounding factors of frailty (including socio-demographic characteristics, lifestyle and behaviors, and chronic conditions) were fully considered in multivariate analysis.

The limitations also must be mentioned. First, the cross-sectional data were used to investigate relationship between meat intake and frailty, implying that no causality could be derived from this study. Actually, non-frail individuals might consume more meat compared to the frail people, suggesting inverse relationship may exist between meat intake and frailty. Second, although the instruments were tested to be valid and reliable, meat consumption and frailty measured through participant's self-report still implies potential misclassifications. Third, red and white meat was not differentiated from each other, which could not allow us to examine meat-frailty relationship by specific type of meat. Fourth, the intake amount of meat was not available. Thus, the association of meat intake amount with frailty also could not be explored. Fifth, energy intake and income, as associated factors of meat consumption, were not considered in analysis due to lack of data. Sixth, information on socio-demographic attributes, lifestyle and behavior, and chronic diseases was gathered via self-report. The potential recall bias may exist too. Therefore, it shall be with particular prudence when interpreting the inverse association between meat consumption and frailty among older adults examined in this study. In future, longitudinal, especially interventional, studies are needed to further examine the potential causal association of meat intake, measured using frequency and amount, with frailty among community-residing older individuals in different countries/territories worldwide.

In conclusion, a inverse relationship may exist between meat intake and frailty among community-dwelling older individuals in regional China. In the context of a rapid population aging and a rising prevalence of frailty worldwide, the present study has particular significance of public health for building the healthy-aging society, suggesting that inadequate intake of meat may be a risk factor of frailty for community-dwelling older adults.

## Data Availability

The original contributions presented in the study are included in the article/supplementary material, further inquiries can be directed to the corresponding authors.
